# Leucine and HMB Differentially Modulate Proteasome System in Skeletal Muscle under Different Sarcopenic Conditions

**DOI:** 10.1371/journal.pone.0076752

**Published:** 2013-10-04

**Authors:** Igor L. Baptista, Willian J. Silva, Guilherme G. Artioli, Joao Paulo L. F. Guilherme, Marcelo L. Leal, Marcelo S. Aoki, Elen H. Miyabara, Anselmo S. Moriscot

**Affiliations:** 1 Department of Anatomy, Institute of Biomedical Sciences, University of Sao Paulo, Sao Paulo, Brazil; 2 School of Physical Education and Sport, University of Sao Paulo, Sao Paulo, Brazil; 3 School of Arts, Science and Humanities (EACH), University of Sao Paulo, Sao Paulo, Brazil; Wageningen University, Netherlands

## Abstract

In the present study we have compared the effects of leucine supplementation and its metabolite β-hydroxy-β-methyl butyrate (HMB) on the ubiquitin-proteasome system and the PI3K/Akt pathway during two distinct atrophic conditions, hindlimb immobilization and dexamethasone treatment. Leucine supplementation was able to minimize the reduction in rat soleus mass driven by immobilization. On the other hand, leucine supplementation was unable to provide protection against soleus mass loss in dexamethasone treated rats. Interestingly, HMB supplementation was unable to provide protection against mass loss in all treatments. While solely fiber type I cross sectional area (CSA) was protected in immobilized soleus of leucine-supplemented rats, none of the fiber types were protected by leucine supplementation in rats under dexamethasone treatment. In addition and in line with muscle mass results, HMB treatment did not attenuate CSA decrease in all fiber types against either immobilization or dexamethasone treatment. While leucine supplementation was able to minimize increased expression of both Mafbx/Atrogin and MuRF1 in immobilized rats, leucine was only able to minimize Mafbx/Atrogin in dexamethasone treated rats. In contrast, HMB was unable to restrain the increase in those atrogenes in immobilized rats, but in dexamethasone treated rats, HMB minimized increased expression of Mafbx/Atrogin. The amount of ubiquitinated proteins, as expected, was increased in immobilized and dexamethasone treated rats and only leucine was able to block this increase in immobilized rats but not in dexamethasone treated rats. Leucine supplementation maintained soleus tetanic peak force in immobilized rats at normal level. On the other hand, HMB treatment failed to maintain tetanic peak force regardless of treatment. The present data suggested that the anti-atrophic effects of leucine are not mediated by its metabolite HMB.

## Introduction

Bed rest, denervation, hind limb unloading, microgravity, immobilization, elevated secretion of catabolic hormones (e.g. glucocorticoids), and pharmacologic treatment (e.g. dexamethasone) might result in considerable muscle wasting. The well known deleterious consequences of these conditions include decreased muscle fiber cross-sectional area and protein content, reduced force and power output, increased fatigability and increased insulin resistance [1,2]. However, the molecular mechanisms responsible for muscle wasting are still not completely understood [3,4].

Much attention has been given to intracellular pathways associated with muscle mass control such as Akt-mTOR, Myostatin and the Ubiquitin-proteasome system (UPS) [5-8]. Evidence suggests that the enhancement of proteolysis in atrophying muscles results mainly from a general activation of the UPS protein degradation [9-11]. In various types of muscle wasting, including Cushing’s syndrome [12], diabetes [13], sepsis [14], cancer cachexia [15], and renal failure [16], muscles exhibit a common series of adaptations, which include increased content of ubiquitin-protein conjugates [17] and of mRNA encoding ubiquitin [18], certain ubiquitination enzymes [13], and multiple proteasome subunits [16,19].

The UPS is an ATP-requiring multienzymatic process that mediates protein degradation by the proteasome. Briefly, prior to degradation a target protein undergoes a three-step process, which covalently links a polyubiquitin chain to the substrate. Three enzymatic components are involved in this process: E1 (ubiquitin-activating enzyme), E2 (Ub-conjugating enzymes) and E3 (ubiquitin protein ligase), which presents substrate recognition sites [20,21]. The ubiquitinated substrate can then be recognized and degraded by the proteasome. In wasting conditions, the genes Mafbx (muscle atrophy F-box) /atrogin-1 and muscle ring finger 1 (MuRF1), both encoding E3 ubiquitin ligases, are up-regulated [9,22], mainly through FOXO transcription factors [23,24]. In addition, the UPS activity is also influenced by a family of enzymes known as deubiquitinating enzymes (DUB’s) [25]. These enzymes can destabilize the covalent bond formed between polyubiquitin chain and substrate [25]. The role of DUBs in skeletal muscle plasticity is elusive; up to date only one study has determined gene expression of DUBs during longitudinal skeletal muscle growth [26].

In addition to mechanical stimuli and hormonal profile, certain nutritional strategies (e.g. amino acids supplementation) also modulate protein turnover in skeletal muscle [27,28]. It is well known that leucine per se can promote an acute increase in protein synthesis; however, its effects upon degradation pathways are still poorly understood. It is known that elevation of mRNA of atrogenes (~3 days after immobilization) and consequently muscle mass loss (clearly detected at 7 days after immobilization) can be minimized by leucine supplementation, although the precise mechanisms remain to be elucidated [29-32]. A previous study showed [32] that leucine feeding in a protein deprivation model minimizes protein catabolism without affecting expression of atrogenes. These authors suggest that the down regulation of the lysosomal pathway activity, rather than UPS, is preferentially involved in the protective effect of a leucine enriched diet [32]. Although the protein deprivation efficiently activates muscle mass degradation pathways, this experimental model is not best designed for investigating the localized effects of disuse on muscle mass since systemic metabolic effects of protein deprivation might play a major role.

Beta-hydroxy-beta-methyl butyrate (HMB) is a metabolite derived from leucine. Under normal conditions ~5% of leucine is converted to HMB and there is experimental evidence supporting that supplementation of this metabolite plays a role in increased performance and muscle hypertrophy. On the other hand the role of HMB in skeletal muscle wasting is still poorly understood. Although HMB has been shown to minimize muscle wasting in cachexia models, no study has addressed the impact of HMB in localized muscle wasting promoted by disuse. Additional effects conferred to HMB include: protection against skeletal muscle injury, minimization of muscle protein degradation, increase in GH-IGF-1 axis activity and modulation of IGF-1 expression in muscle, leading to enhancement of mTOR pathway activity (See Zanchi et al. [33] for review) [34]. Although it has been shown that HMB stimulates myogenic differentiation and survival via PI3K/AKT pathway [35], it is still unclear whether HMB could modulate this pathway under atrophic conditions.

Our hypothesis contemplates that HMB could play a contributive role in the anti-atrophic effects of leucine, therefore we have compared the effects of leucine and HMB during hind limb immobilization and dexamethasone treatment, representing respectively localized and systemic models of muscle wasting. The results showed a specific protective effect of leucine during hind limb immobilization and no effect of HMB in both hind limb immobilization and dexamethasone treatment, pointing that HMB does not mediate the anti-atrophic effects of leucine. Furthermore, this study indicates the idea that minimization of the UPS activation is a major player in protection against atrophy in the dexamethasone/immobilization models.

## Materials and Methods

The protocols used in this study are in agreement with ethical principles in animal research followed by the Brazilian College of Animal Experimentation and were approved by the Institute of Biomedical Sciences / University of São Paulo - Ethical Committee for Animal Research (# 151/12).

### Animals and supplementations

Male Wistar rats (n=256, n=8 per group) (~2 months-old) weighing 260±25 g were housed in standard plastic cages in an animal room with controlled environmental conditions and maintained on standard food and water *ad libitum*.

Leucine (L-Leucine, Ajinomoto^©^, Tokyo, Japan) was orally administered (*via gavage*) once a day in a dose of 2.7 g/kg body mass per day (modified from Kimball and Jefferson [36]), starting 3 days prior to immobilization or dexamethasone treatment. A control group received saline only.

HMB (Calcium β-Hydroxy-β-Methylbutirate, Lonza^©^, Basel, Switzerland) was orally administered (*via gavage*) once a day in a dose of 600mg/kg/day, starting 3 days prior to immobilization or dexamethasone treatment. A control group received saline only.

The supplementations effects per se were analyzed using animals supplemented for 1, 4 and 6 days without atrophic conditions and pre-charge.

### Immobilization procedure and dexamethasone treatment

Monolateral hind limb immobilization was performed in the left hind limb by applying a cast with total plantar extension, knee was kept in total extension, as previously reported [29,37].

Eight groups of animals were submitted to atrophic conditions and analyzed 1, 2, 3 and 7 days after immobilization (Imob 1d, 2d, 3d and 7d) or dexamethasone treatment (Dexa 1d, 2d, 3d and 7d). Another eight groups received leucine or HMB starting 3 days prior to immobilization which was maintained up to the end of the experiment (Imob 1d+Leu, Imob 2d+Leu, Imob 3d+Leu and Imob 7d+Leu ; Imob 1d+HMB, Imob 2d+HMB, Imob 3d+HMB; Imob 7d+HMB). To analyze the effect of leucine and HMB under hormonal atrophy, eight groups received leucine or HMB starting 3 days prior to dexamethasone treatment which was maintained up to the end of the experiment (Dexa 1d+Leu, Dexa 2d+Leu, Dexa 3d+Leu and Dexa 7d+Leu ; Dexa 1d+HMB, Dexa 2d+HMB, Dexa 3d+HMB; Dexa 7d+HMB).

### Tissue samples

Soleus muscles were exposed and used for in vivo contraction assays and after that were removed and weighed. Subsequently, these muscles were transversely cut in half; one segment was immersed in cold isopentane for 30 seconds, cooled in liquid nitrogen and stored at -70°C for histochemistry. The other segment was snap frozen in liquid nitrogen and stored at -70°C for RNA and protein expression analysis.

### Histochemistry and cross-sectional area

The frozen muscles were cut into 10-µm cross-sections through the proximal to distal region using a cryostat (Leica^©^ CM3050, Nussloch, Germany). Alternate serial cross-sections were obtained in the proximal and middle regions of both muscles incubated for myofibrillar ATPase activity after alkali (ATPase, pH 10.3) or acid pre-incubation (ATPase, pH 4.3) [38,39]. After classification, the cross-section area was obtained from approximately 2000 muscle fibers and shown as mean±s.d. The cross-sectional area was determined using a microscope (Nikon Eclipse E600^©^, Fukuoka, Japan) equipped with a digital video camera and image software (Metamorph®, Universal Imaging Corporation^©^, Downingtown, USA).

### In vivo contraction assays

All groups submitted to immobilization or dexamethasone treatment for 7 days were tested for muscle function prior to euthanasia. With the animal sedated, the distal tendon of the soleus muscle of the right hind limb was exposed and attached to the lever arm of a servomotor (BIOPAC Systems, Goleta, CA, USA). The knee of the right hind limb was fixed and then muscle twitch contractions were induced by electrical stimulation of the sciatic nerve. The muscle length was adjusted to produce a maximum twitch force in a single twitch (stimulation at 4 V). The muscle length that produced the maximum twitch force was considered the optimum muscle length. With the muscle in optimum length, tetanic force was induced at a stimulation frequency of 250 Hz at 4 V. Data were collected using Acknowledge software (BIOPAC Systems, version 3.9.1.6 for Windows).

### RNA isolation

Muscle samples (25 mg) were homogenized with a Polytron^©^ (Kinematica AG, Littau-Lucerne, Switzerland) and total RNA was isolated using the Trizol reagent (Invitrogen^©^, Carlsbad, USA) following the manufacturer’s recommendations. These samples were dissolved in free ultra-filtered water, and their concentrations were determined by measuring the optical density at 260 nm with an Eppendorf^©^ spectrophotometer (Eppendorf^©^, Hamburg, Germany). The purity of the RNA was determined by calculating the absorbance ratio at 260 nm and 280 nm and RNA integrity was checked on a 1% agarose gel stained with ethidium bromide.

### Reverse transcription (RT) reaction

One µg of the total RNA was used in a reaction containing oligo dT (500 µg/ml), 10 mM of each dNTP, 5X First-Strand Buffer, 0.1 M DTT and 200 U of reverse transcriptase (SuperScript II-Invitrogen^©^, San Diego, USA). RT reaction was performed at 70 °C for 10 min followed by 42 °C for 60 min and 10 min at 95 °C.

### Oligonucleotide primers

Primer sets for rat Mafbx/Atrogin-1(Foward-TACTAAGGAGCGCCATGGATACT, Reverse- GTTGAATCTTCTGGAATCCAGGAT), UBP45 (Forward-CAGCATGCGTACCTCCTACACC, Reverse- ACTCTTTGAATTCTTGGCTTTGTTGA), UBP69 (Forward- CCGGACACAGCCCATGAG, Reverse-GTAGCGGGACGATTCTGTATAGC), USP28 (Forward- AAAGGCCAGTAATGGTGACATCA, Reverse-GTCGTGACTGGGCTCCTTAACT) and Cyclophilin A (Forward-GCCGATGACGAGCCCTTG, Reverse-TGCCGCCAGTGCCATTAT) were designed using the Primer Express Software 2.0 (Applied Biosystems^©^, Foster City, USA). Primer sequences for Murf-1 (Forward-TGACCAAGGAAAACAGCCACCAG, Reverse-TCACTCCTTCTTCTCGTCCAGGATGG) were obtained from Wray et al. [40]. All primers were synthesized by IDT^©^ (Coralville, USA).

### Quantitative polymerase chain reaction (PCR)

For each gene, PCR was performed in duplicate with a 25 µl reaction volume of 5-20 ng of cDNA, 12.5 µl Syber Green Master Mix^©^ (Applied Biosystems^©^) and 50-200 nM of each primer. PCR analyses were carried out using the following cycle parameters: 50°C for 2 min, 95°C for 10 min, followed by 40 cycles of 95°C for 15 s, and 60°C for 1 min. The fluorescence intensity was quantified and amplification plots were analyzed with a Corbett RotorGene 6000 (Qiagen^©^, Hilden, Germany). Results were expressed using the comparative cycle threshold (CT) method as described in the User Bulletin 2 from the manufacturer (Applied Biosystems^©^, Foster City, USA). ΔCt values were calculated in every sample for each gene of interest as follows: Ct_gene of interest_ - Ct_reporter gene_; with Cyclophilin A as the control gene. Relative changes in the expression level of one specific gene (ΔΔCt) were calculated by subtraction of the ΔCt from the control group (used as a calibrator) to the corresponding ΔCt from the treated groups.

### Western blotting analysis

The primary antibodies used for Western blotting were: rabbit polyclonal antibodies against ubiquitin (1:1,000; cat# A-100; BostonBiochem^©^, Cambridge, USA), PI3K (1:1,000; cat# 4255; Cell Signaling), AKT (1:1,000; cat# 9272; Cell Signaling), phospho-AKT Thr308 (1:1,000; cat# 4056S; Cell Signaling), phospho-AKT Ser473 (1:1,000; cat# 4058S; Cell Signaling), 4E-BP1 (1:1,000; cat# 9644; Cell Signaling) and monoclonal mouse antibody against Sarcomeric Actin (1:1,000; cat# M0874; Dako^©^, Glostrup, Denmark). The secondary antibodies used were alkaline-phosphatase conjugate goat anti-rabbit IgG (1:1,000; cat# D0487; Dako^©^, Glostrup, Denmark) and rabbit anti-mouse IgG (1:1000; cat# D0314; Dako^©^, Glostrup, Denmark).

Soleus muscles were homogenized in an extraction solubilization buffer (50 mM potassium phosphate buffer (pH 7.0), 0.3 M sucrose, 0.5 mM DTT, 1 mM EDTA (pH 8.0), 0.3 mM PMSF, 10 mM NaF), and phosphatase inhibitor cocktail (1:100; Sigma-Aldrich^©^, St. Louis, USA) or RIPA buffer (0.625% Nonidet P-40, 0.625% sodium deoxycholate, 6.25 mM sodium phosphate, 1 mM EDTA pH 7.4) containing 10 µg/ml of protease inhibitor cocktail (Sigma-Aldrich^©^, St. Louis, USA) in order to detect the expression of ubiquitin conjugating protein. Homogenates were centrifuged at 10,000*g* for 10 min at 4°C and the supernatant used. Protein concentration was determined by Bradford assay (Bio-Rad^©^, Hercules, USA) with bovine serum albumin as standard.

Equal amounts of protein (50 µg) were run on 12% SDS-PAGE and transferred to a nitrocelulose membrane (Biorad^©^, Hercules, USA). The membranes were stained with Ponceau S to confirm the protein amount and then rinsed with Tween Tris-buffered saline solution (0.5 M NaCl, 50 mM Tris-HCl, pH 7.4 and 0.1% Tween 20). All membranes were incubated with 0.1% Tween 20 in 0.1 M phosphate-buffered saline (PBS, pH 7.4) at room temperature. Membranes were then incubated overnight with primary antibodies at 4°C. After a 15-min wash in Tween Tris-buffered saline solution, membranes were incubated with alkaline-phosphatase (AP) conjugated secondary antibody for 1 hour at room temperature and washed again for 15 min in Tween Tris-buffered saline solution. After washing, speciﬁc bands were visualized by enzymatic colorimetric NBT/BCIP (Roche, Mannheim, Germany). Loading variations were monitored by sarcomeric actin Western immunoblotting [41].

### Statistical analysis

Multiple comparisons of mean values were performed using analysis of variance (ANOVA) and a post-hoc Tukey’s test to compare mean values when appropriate. For all comparisons, a *p*<0.05 was considered significant.

## Results

**Figure 1:**
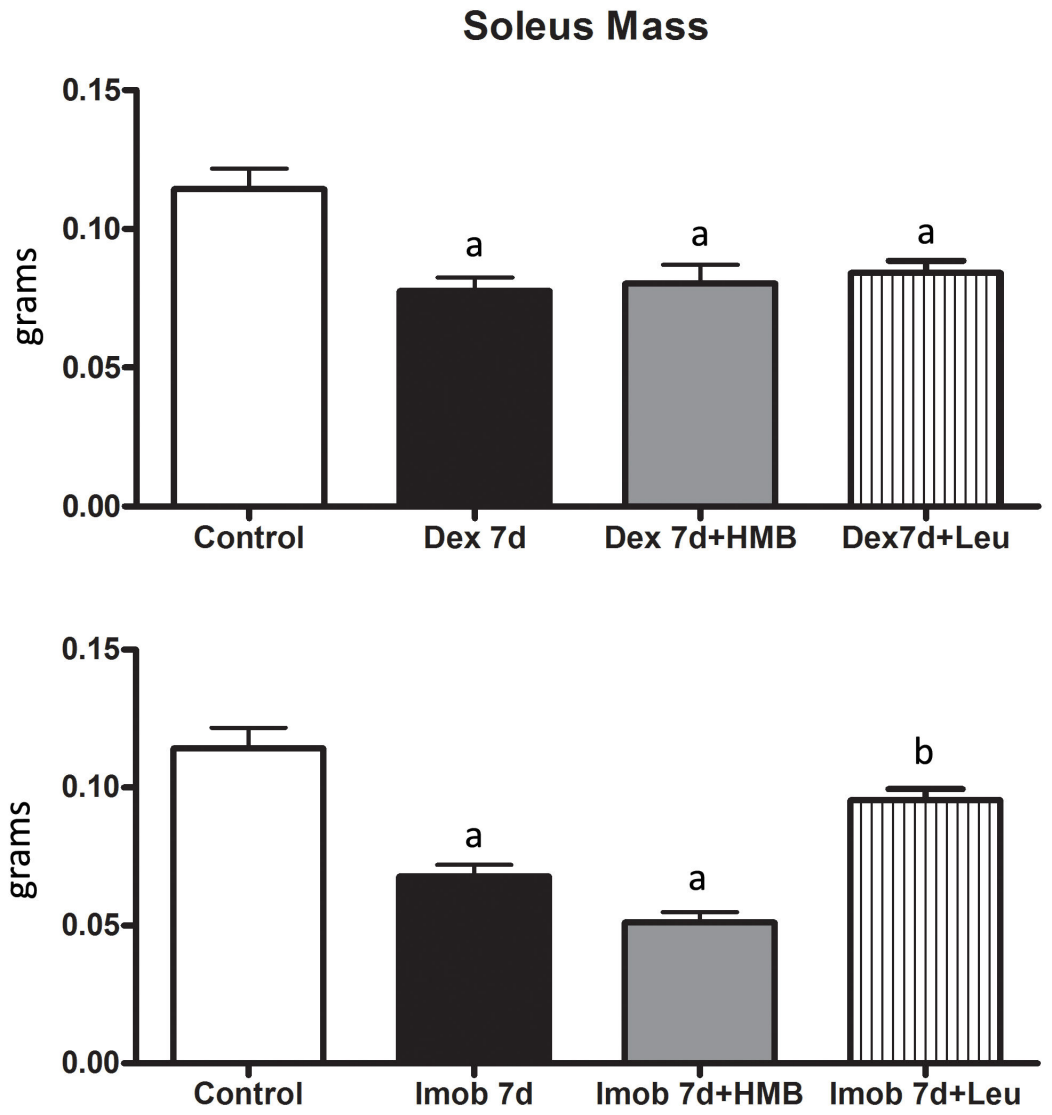
Soleus muscle mass. Soleus muscle mass (grams) 7 days post either hormonal treatment (Dexa 7d; A) or hind limb immobilization (Imob 7d; B). Rats were supplemented with either HMB (Dexa 7d+HMB; A and Imob 7d+HMB; B) or Leucine (Dexa 7d+Leu; A and Imob 7d+Leu; B). a, p<0.05 vs control, b - p<0.05 vs. Imob. Bars represent mean+SD.

The first aim of the present study was to compare the impact of HMB and leucine treatment upon skeletal muscle mass in two wasting models, dexamethasone and hind limb immobilization. One week under dexamethasone treatment, as expected, caused about 35% soleus muscle mass loss, interestingly administration of either HMB or leucine was unable to change the effect of dexamethasone (Figure 1A). As expected, immobilization induced about 40% soleus muscle mass loss and in contrast to the dexamethasone model, we found differential protective effects of HMB and leucine. While HMB was unable to slow down soleus muscle mass loss, leucine was able to keep soleus muscle mass loss at similar levels when compared to control group (Figure 1B).

**Figure 2:**
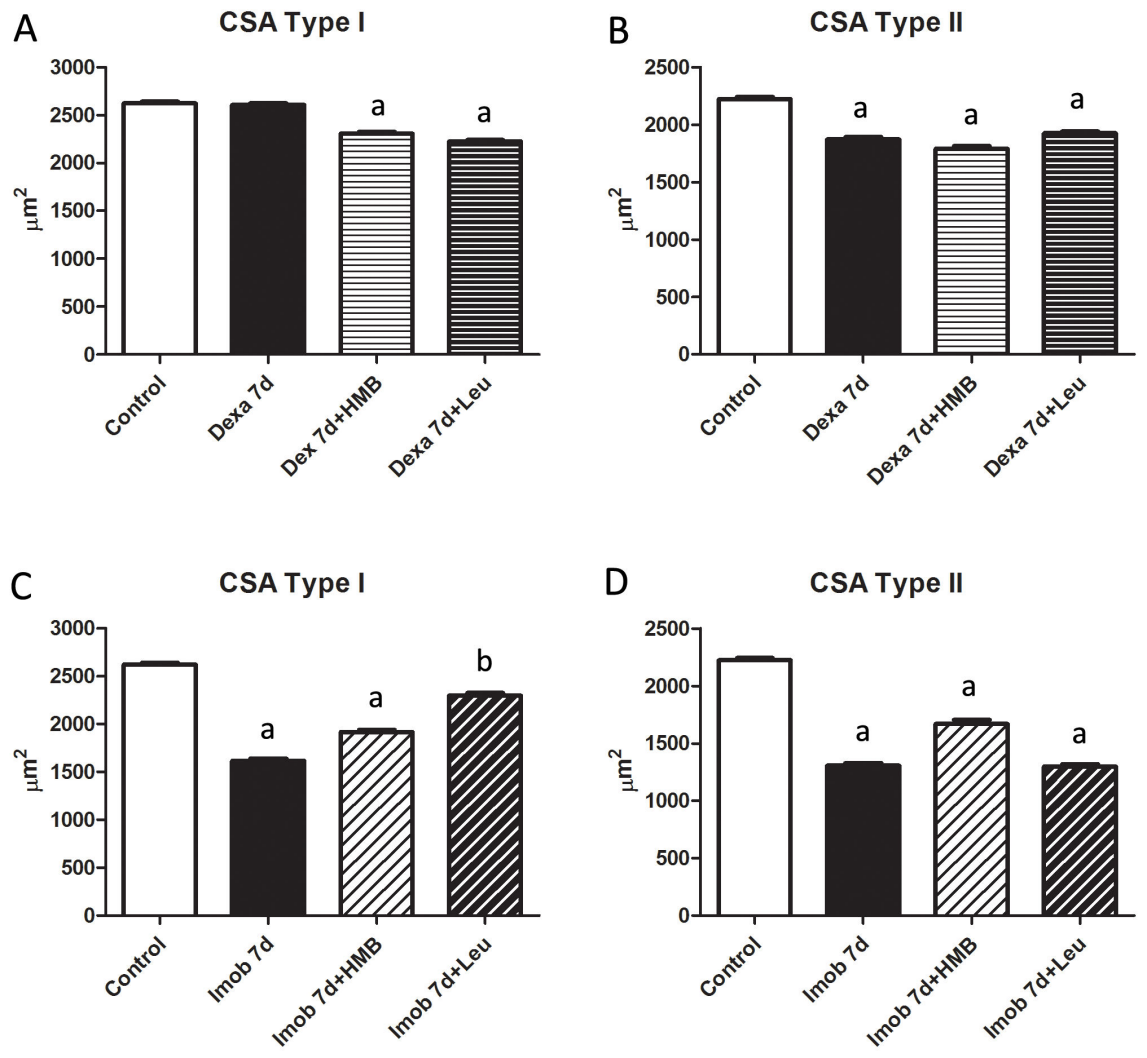
Cross sectional area from fiber types I and II. Soleus muscle fibers cross sectional area (CSA, µm^2^) of fiber types I and II 7 days post either dexamethasone treatment (A and B) or hind limb immobilization (C and D). a- p<0.05 vs. Control; b - p<0.05 vs. Imob. Bars represent Mean+S.D.

In order to get further insight on the effects of HMB and leucine upon skeletal muscle under atrophy, the cross sectional area of specific fiber types was determined. Under our experimental conditions, dexamethasone promoted a drop in the cross sectional area only in type II fibers. In addition neither HMB nor leucine were able to counteract this effect (Figure 2A and B). Immobilization also promoted a decrease in the cross sectional area of type I and type II muscle fibers, interestingly leucine but not HMB was able to protect type I muscle fibers from cross sectional area loss. Neither leucine nor HMB were able to protect type II fibers against cross sectional area loss (Figure 2D).

**Figure 3:**
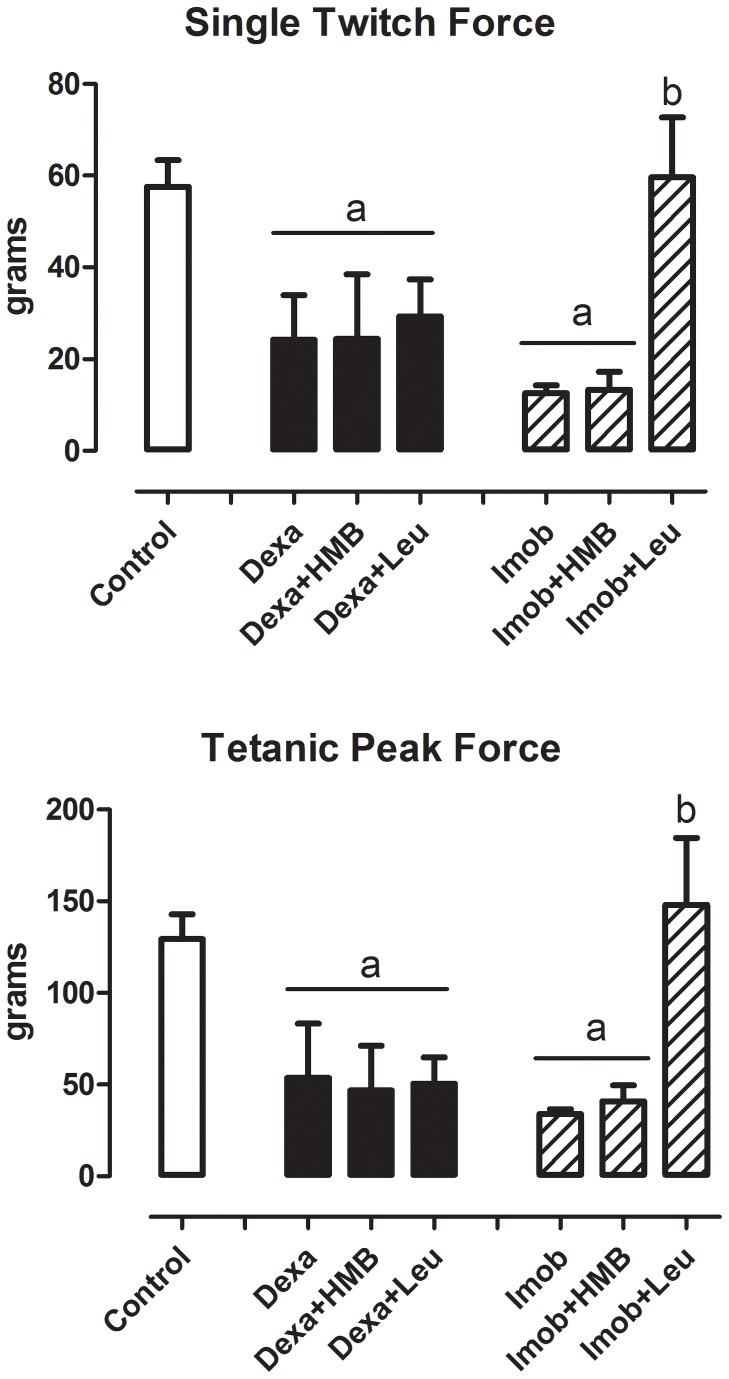
Effect of HMB and leucine supplementation under single twitch and tetanic force during atrophic stimuli. Single twitch force (in grams) and Tetanic peak force (in grams) in animals submitted to dexamethasone treatment or hind limb immobilization. White bars represent Mean±SD in Control group; black bars represent Mean±SD in Dexa, Dexa+HMB and Dexa+Leu groups; Striped bars represent Mean±SD in Imob, Imob+HMB and Imob+Leu groups. a- p<0.05 vs. Control; b - p<0.05 vs. Imob.

In addition to morphological measurements, the muscle function after leucine and HMB treatments was investigated in rats submitted to dexamethasone and immobilization. As expected, both dexamethasone and immobilization induced a severe drop (~70%) in single twitch and tetanic force (Figure 3). While HMB was completely ineffective to restore muscle force, leucine was able to fully revert the decrease in muscle force driven by immobilization, but not by dexamethasone treatment (Figure 3).

**Figure 4:**
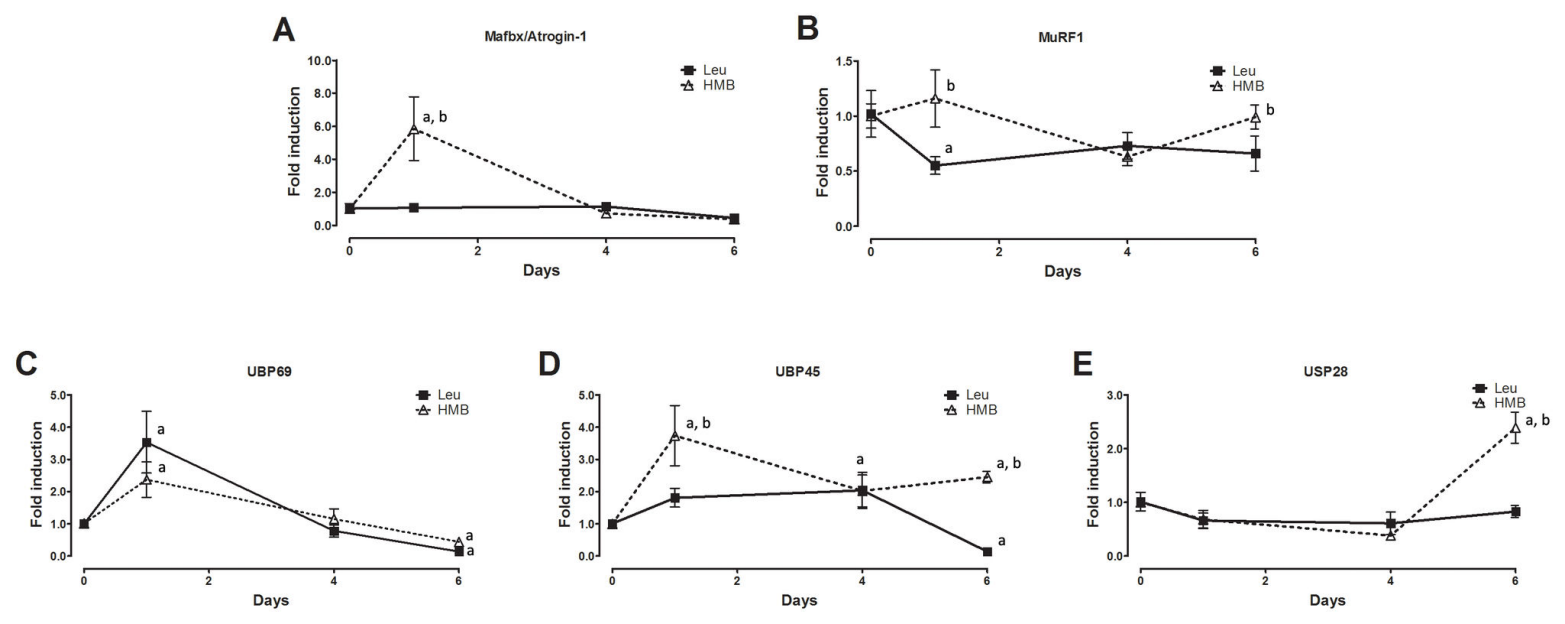
Atrogenes and deubiquitinating enzymes gene expression during HMB or leucine supplementation per se. Gene expression of Atrogin-1 (A), MuRF1 (B), UBP69 (C), UBP45 (D) and USP28 (E) during leucine (filled line -1, 4 and 6 days) or HMB (dashed line -1, 4 and 6 days) supplementation. Control is arbitrarily set to 1. Data are expressed as mean±S.D. (n=5 per group). a- p<0.05 vs. Control; b - p<0.05 vs. Leu.

**Figure 5:**
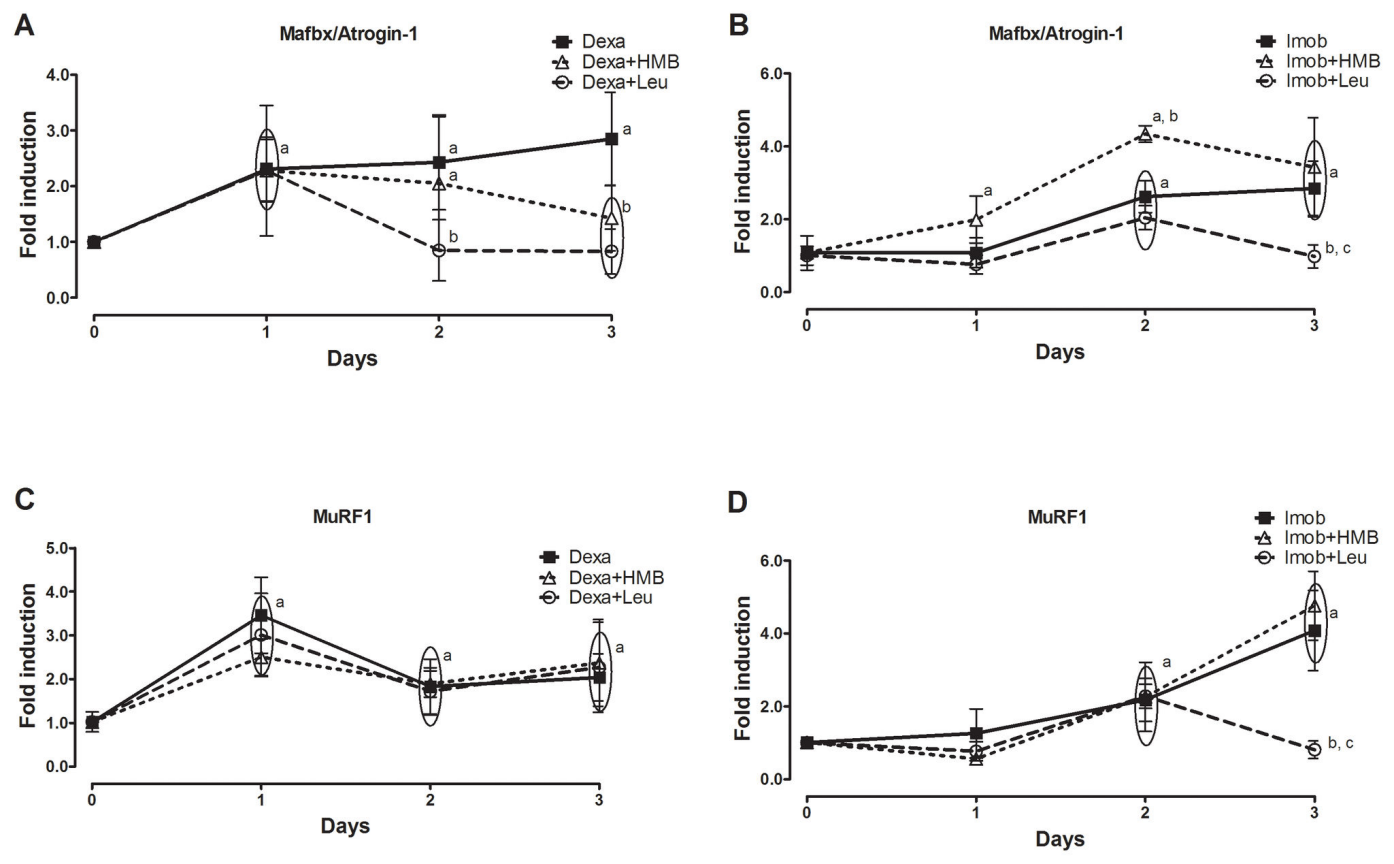
Effect of HMB and leucine supplementation under E3 ligases gene expression during atrophic stimuli. Gene expression of Atrogin-1 (A and B) and MuRF1 (C and D) during either dexamethasone treatment (A and C) or hind limb immobilization (B and D). Control is arbitrarily set to 1. Data are expressed as mean±S.D. (n=5 per group). a- p<0.05 vs. Control; b - p<0.05 vs Dexa or Imob in respective time point; c - p<0.05 vs. Imob+HMB in respective time point.

**Figure 6:**
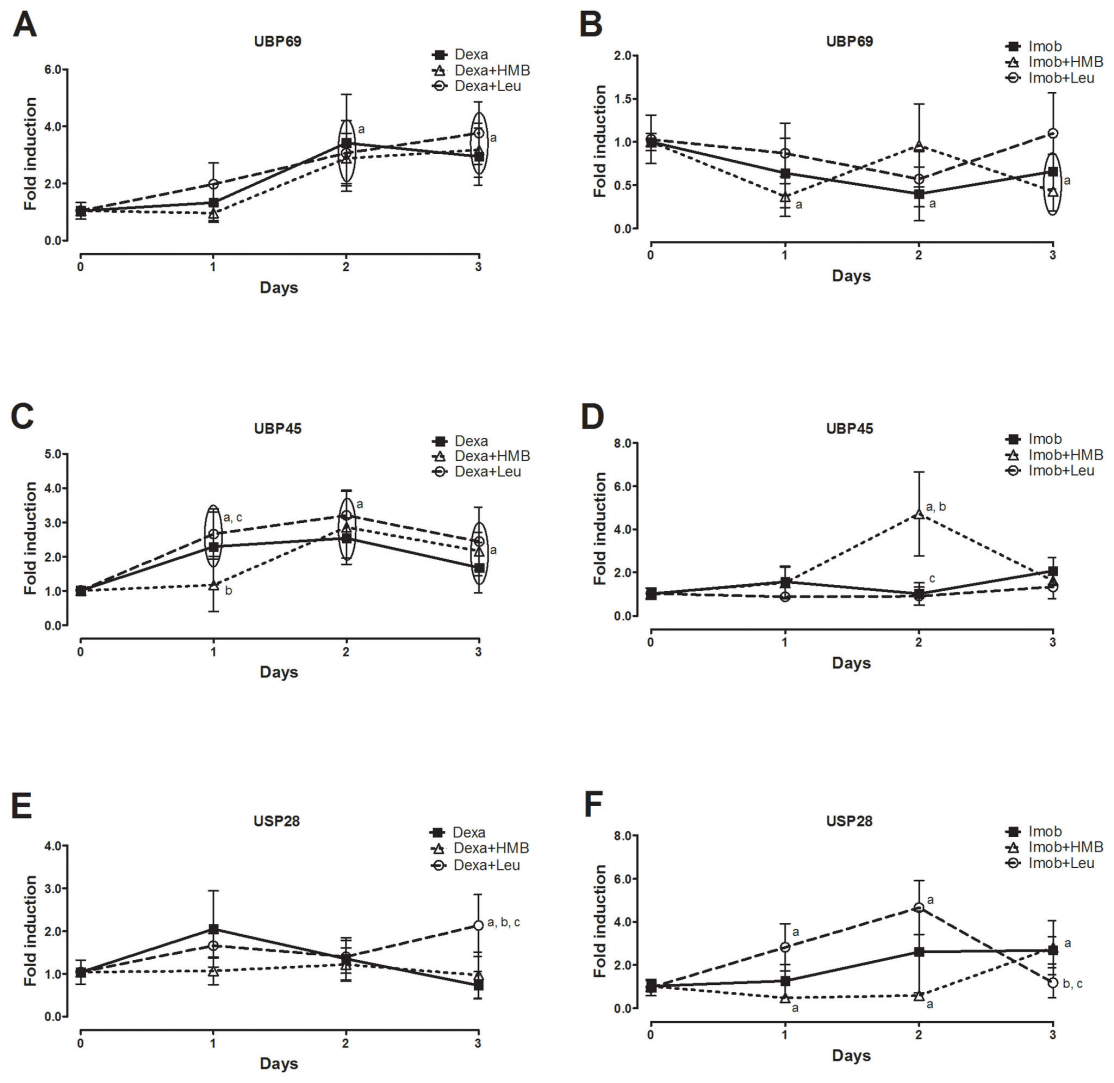
Effect of HMB and leucine supplementation under deubiquitinating enzymes gene expression during atrophic stimuli. Gene expression of UBP69 (A and B), UBP45 (C and D) and USP28 (E and F) during either dexamethasone treatment (A, C and E) or hind limb immobilization (B, D and F). Control is arbitrarily set to 1. Data are expressed as mean±S.D. (n=5 per group). a- p<0.05 vs. Control; b - p<0.05 vs Dexa or Imob in respective time point; c - p<0.05 vs. Dexa+HMB or Imob+HMB in respective time point.

Next gene expression of E3 ligases and deubiquitinases in rats submitted to dexamethasone and immobilization was evaluated. The two molecular hallmarks of atrophic state Mafbx/Atrogin-1 and MuRF1 were differentially regulated by leucine and HMB per se (Figure 4A and B). While leucine per se was overall not able to strongly stimulate expression of neither one of those genes, HMB increased (~6 fold) expression of Mafbx/Atrogin-1 peaking at the 1^st^ day of treatment and returning to basal levels at 4^th^ day of treatment (Figure 4A). The deubiquitinases were also differentially expressed by leucine and HMB. UBP 69 was up regulated by both leucine (~3.5 fold) and HMB (~2.5 fold) at short periods of supplementation (Figure 4C). While UBP 45 gene expression was down regulated by leucine (~0.1 fold), it was up regulated by HMB (~4 fold, Figure 4D). USP 28 gene expression was unaffected by leucine and up regulated by HMB (~2.5 fold, Figure 4F). After depicting the effect of leucine and HMB per se, we investigated the impact of those in rats under dexamethasone and immobilization. For that purpose, rats were preloaded with either HMB or Leucine for 3 days in order to assure full effectiveness and also to set the dexamethasone/immobilization treatment in a time frame where effects of HMB and Leucine per se were minimized (time zero in Figures 5 and 6 is equivalent to 3 days of treatment in Figure 3). Dexamethasone increased Mafbx/Atrogin-1 and MuRF1 gene expression (2-3 fold) at 1^st^ day of treatment regardless of either HMB or leucine administration (Figure 5A and C). At second and third days, on the other hand, leucine was able to return Mafbx/Atrogin-1 gene expression to control levels. Differently, HMB was able to return Mafbx/Atrogin-1 to control levels only on the 3^rd^ day of dexamethasone treatment (Figure 5A). Neither leucine nor HMB were able to decrease the positive effect of dexamethasone upon MuRF1 gene expression (Figure 5C). The gene expression of both Mafbx/Atrogin-1 and MuRF1 increased up to 2^nd^ to 3^rd^ days of immobilization (Figure 5B and D). Leucine, but not HMB, was able to return Mafbx/Atrogin-1 and MuRF1 to control levels on the 3^rd^ day of immobilization (Figure 5B and D). It is noticeable, however, that unexpectedly HMB treatment enhanced MuRF1 gene expression in immobilized muscles when compared to control (Figure 5B).

Regarding deubiquitinases, the impact of leucine and HMB upon dexamethasone treated and immobilized rats was rather minor (Figure 6). Nonetheless, HMB was able to increase UBP45 gene expression in rats on the 2^nd^ day of immobilization (Figure 6D). Leucine was able to increased gene expression of USP28 in dexamethasone treated animals (Figure 6E). Also leucine was able to increase USP28 gene expression on the 2^nd^ and 3^rd^ days of immobilization (Figure 6F).

**Figure 7:**
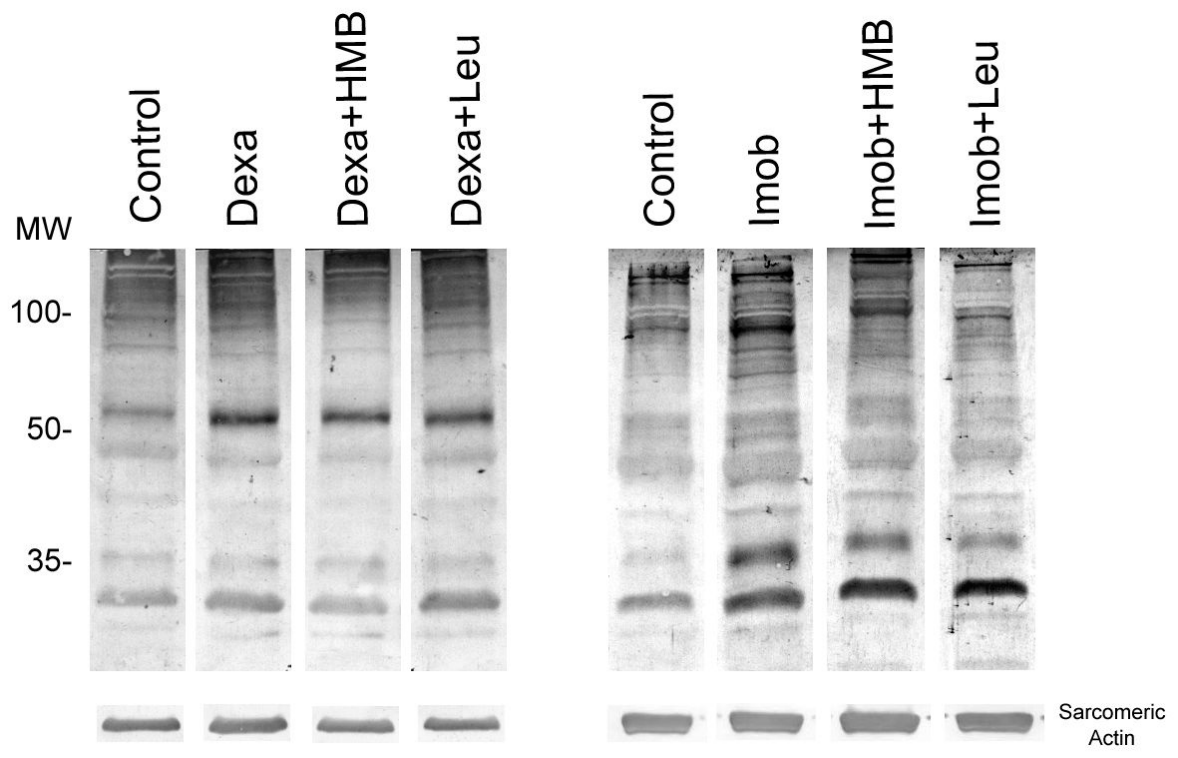
Total ubiquitinated proteins levels. Representative Western blot of ubiquitin-protein conjugates 3 days after dexamethasone treatment or hind limb immobilization, under HMB (Dexa+HMB and Imob+HMB, respectively) or leucine supplementation (Dexa+Leu and Imob+Leu, respectively). Each lane represents one of three independent experiments (n=3 per group).

In order to understand the overall state of ubiquitination, an antibody, which detects mono and poliubiquitinated proteins, was utilized. The results show that as expected, dexamethasone increases the abundance of ubiquitinated proteins (Figure 7). Interestingly, neither leucine, nor HMB were able to counteract this effect. Immobilized animals also showed elevated levels of ubiquitinated proteins (Figure 7). While Leucine treatment effectively counteracted the increase in ubiquitinated proteins induced by immobilization, HMB partially decreased ubiquitination levels as compared to control (Figure 7).

**Figure 8:**
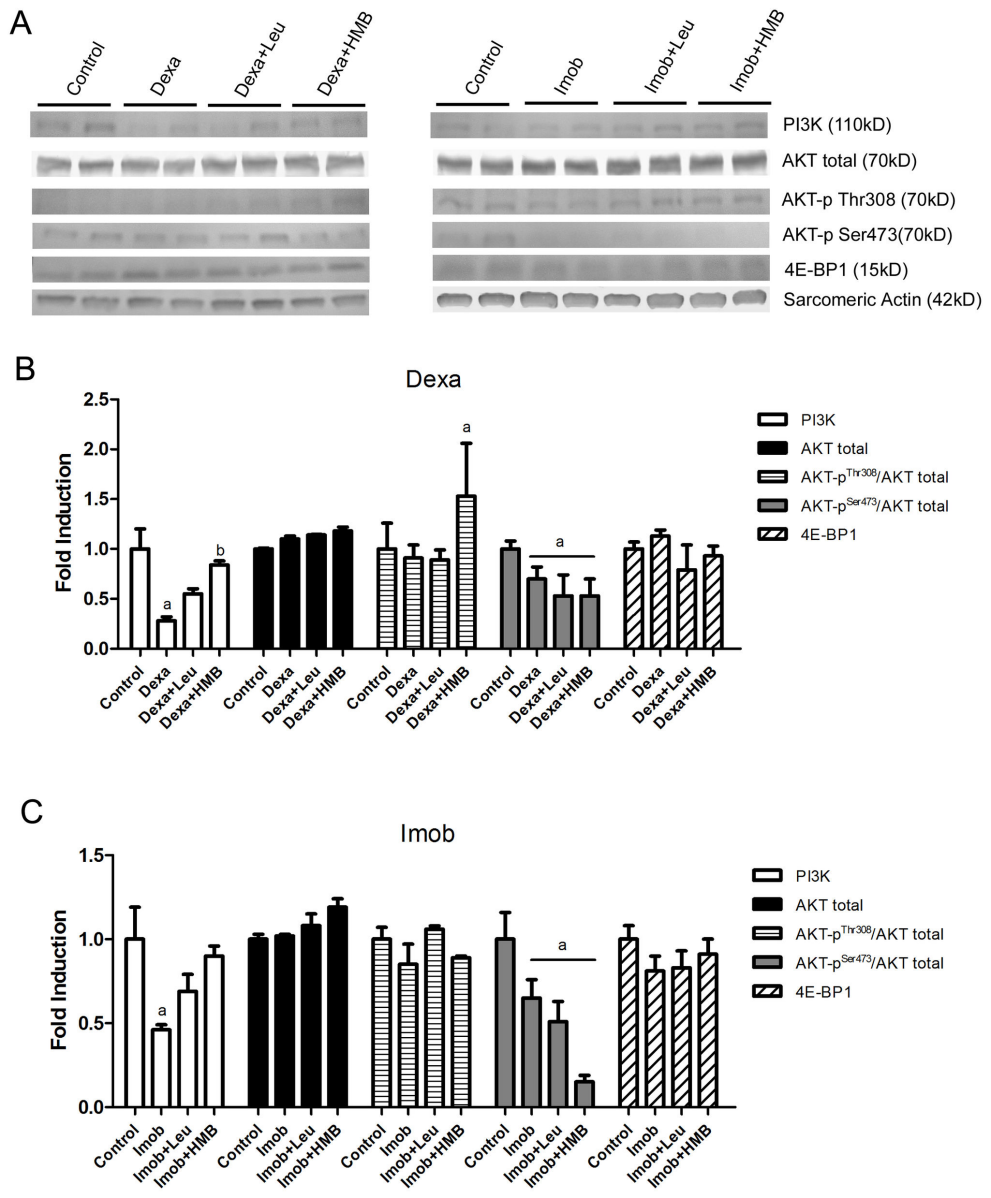
Effect of HMB and leucine under PI3K/AKT protein levels during atrophic stimuli. Representative protein level of PI3K, AKT total, AKT phosphorilation residues at Thr308 and Ser473 and 4E-BP1, 3 days after dexamethasone administration or hind limb immobilization, under leucine (Dexa+Leu and Imob+Leu, respectively) or HMB (Dexa+HMB and Imob+HMB, respectively). Each pair of lanes represents a duplicate of each group (n=4 per group). Sarcomeric actin was used as loading control. The bars in B and C represent mean±S.D. a -p<0,05 vs. Control.

In an attempt to clarify whether leucine and HMB activate the PI3K/AKT pathway during atrophic conditions, the levels of key proteins on this pathway were analyzed. The results showed a strong decrease on PI3K levels during dexamethasone administration and immobilization (p<0,05 vs. Control, Figure 8A, B and C). Interestingly, under leucine or HMB treatments, the effect of dexamethasone administration and immobilization upon PI3K levels was attenuated. The levels of total AKT were not altered by either dexamethasone or immobilization and AKT phosphorylation at Thr308 was not affected by both dexamethasone and leucine. On the other hand, HMB was able to increase Thr308 phospho-AKT levels in dexamethasone treated animals (p<0.05 vs. Control, Figure 8A and B). AKT phosphorilation at Ser473 was reduced by dexamethasone/immobilization and remained reduced despite of either leucine or HMB supplementation (p<0.05 vs Control, Figure 8A, B and C). 4E-BP1 levels were unchanged by immobilization/dexamethasone and also unresponsive to either leucine or HMB (Figure 8).

## Discussion

In this study, we have shown that HMB is not able to provide protection against skeletal muscle wasting in rats submitted to two different atrophy inducing models, dexamethasone treatment and immobilization. In contrast, leucine acts as an efficient protector only in the immobilization model, acting on type I fibers.

HMB is an important metabolite derived from leucine and about 5% of leucine is endogenously converted to HMB [42]. Although previous studies have shown that HMB can exert important effects upon skeletal muscle [43,44], no studies have systematically compared the effects of HMB and leucine in skeletal muscle under catabolic conditions. Such a study is of high interest to understand the individual effects of HMB, isolating on what is leucine dependent and HMB dependent regarding skeletal muscle trophicity. In this sense, the present study complements a previous study [29], which showed that leucine supplementation successfully protects skeletal muscle against wasting in rats, submitted to immobilized hind limb. At that point, we were not able to determine whether HMB could also act as an anti-wasting agent. Herein, the results of the present study clearly indicate that HMB does not play a role in the skeletal muscle anti-wasting effect driven by leucine supplementation since 1) HMB does not exert protection against skeletal muscle mass loss as leucine in immobilized animals, 2) HMB does not minimize the rise in atrogenes gene expression in immobilized animals and 3) HMB does not minimize loss of force in immobilized animals. Interestingly, a recent study has pointed out that HMB supplementation does improve skeletal muscle performance i.e. tetanic force, although the authors have not detected increases in skeletal muscle mass [44]. These results combined with the results of the present study emphasize the notion that the hypertrophic and atrophic states are not the result of a continuum controlled by the same pathways, those states seem to be also under the control of different mechanisms. It should be noted though, that in the study of Pinheiro et al. [44], a different dose and time of treatment were employed as compared to the present study. Nonetheless it is possible to envision that HMB would be more related to activating pro-hypertrophic/anabolic pathways, rather than inhibiting pro-atrophic/catabolic ones.

Previous studies have addressed the effects of HMB in skeletal muscle mass. Hao et al. [45] showed that HMB in a hind limb suspension model, in line with our observations, is unable to prevent atrophy (i.e. skeletal muscle mass). On the other hand, in contrast with the results presented herein, the same authors detected that HMB provides a protective effect upon fiber cross sectional area and isometric force loss induced by hind limb suspension (Hao et al. 2011). This controversy might be related to response variation in different species (Wistar Rats in present study vs Fisher 344x Brown Norway rats in Hao et al. study [45]) and age (2 month old animals in the present study vs 34 month olds in Hao et al. study [45]). It is widely recognized that skeletal muscle atrophy is much more severe in elders, and that certain mechanisms play a more important role, such as apoptosis. In fact Hao et al. [45] found that apoptosis is significantly minimized in atrophying HMB supplemented animals. Therefore, it is possible to consider that HMB could be more effective in elder rats, acting throughout minimization of apoptosis. A recent study using L6 cells, showed that HMB is able to minimize the increase in atrogenes induced by dexamethasone, nonetheless, no in vivo experiments were conducted [43]. These previous studies along with the present investigation foresee additional work to pinpoint the effectiveness of HMB as an anti-atrophic agent.

HMB per se, exerts surprising effects in non-immobilized muscles. Interestingly, Mafbx/Atrogin-1 gene expression is sharply induced (~6 fold) by HMB, while leucine has no effect (Figure 4). Although not responsive to HMB, MuRF1, as one could expect, is down regulated by leucine per se (~40%) in intact muscles, clearly showing differential effects. It is not possible to point out the biological meaning of this strong positive effect of HMB upon Mafbx/Atrogin-1 in intact muscles. Several studies have shown that HMB does not drive atrophy [43,44], actually, it is clearly related with ergogenic effects [46]. We speculate that the strong rise in Mafbx/Atrogin-1 driven by HMB might be counteracted by a rise in deubiquitinases (Figure 4). Another possibility contemplates a compensatory down regulation of other elements of the proteasome system such as E2 and proteasome subunits [47,48].

While in the present study, both dexamethasone and immobilization consistently boosted mRNA levels of atrogenes, deubiquitinases did not seem to follow a pattern. For example, UBP69 gene expression was up regulated in dexamethasone treated animals and down regulated in immobilization (Figure 6). UBP 45 gene expression was elevated in dexamethasone treated animals and unaltered in immobilization. These data suggest that those genes might not be quite linked to an atrophying program and might be more related to other cellular processes.

One of the key findings of the present study regards the differential effects of leucine upon atrophying skeletal muscle under dexamethasone and immobilization regimens. Leucine supplementation clearly is a potent anti-wasting agent in immobilized muscles, on the other hand rather ineffective in animals under dexamethasone regimen. Considering it is known that leucine acts through minimizing the increase in atrogenes during atrophy in the immobilization model but does not accomplish this effect in dexamethasone treated animals, one could interpret that the immobilization and dexamethasone might include modulation of atrogenes throughout different mechanisms. Regarding FOXO activity previous studies indicate that FOXO1 and FOXO3a seem to be similarly activated (dephosphorylated) by both dexamethasone and immobilization [49,50]. Another possibility includes that co-factors able to modulate FOXO transactivation capacity could be targeted by leucine in the immobilization condition and not in the dexamethasone condition. A candidate for such a function is for example p300; which is an acetyltransferase known to inhibit FOXO [51]. Other candidates could be Stress-Activated Protein Kinases (SAPKs). Those factors are known to transfer FOXO3a from nucleus to cytoplasm [52]. Other possibilities include differential effects of immobilization and dexamethasone in FOXO independent pathways such as NF-κB [53,54], which are also able to trigger atrogenes.

We have observed that dexamethasone drives loss of mass, which is not followed by a mirrored loss of muscle fiber CSA while in the immobilization model there is a good correlation between mass loss and CSA drop. Although we do not have a precise explanation for this difference in response, it is possible that the acute systemic effects of dexamethasone play a role, i.e. potent diuresis and natriuresis [55], resulting in muscle dehydration.

Another key question not yet answered raised by the present study is how leucine is able to minimize the increased atrogenes expression driven by immobilization. One possibility contemplates a direct effect of leucine upon PI3K/AKT. In fact, it has been previously shown that leucine can directly activate mTOR, a downstream molecule [27,56], suggesting that leucine could modulate this pathway. Our results clearly show that leucine is able to minimize the down regulation of certain elements of PI3K/AKT pathway during atrophy induced by dexamethasone treatment and immobilization, such as PI3K. On the other hand downstream elements of this pathways evaluated in the present study were not influenced by leucine treatment, suggesting that the decrease in PI3K/AKT pathway activity driven by immobilization cannot be mitigated by leucine. In fact, in a previous study we observed that immobilized rats supplemented with leucine do not exhibit increased protein synthesis rate as compared to immobilization alone [29], suggesting that the effects of leucine upon the PI3K/AKT pathway observed in the present study do not reflect in increased protein synthesis. It is not to be excluded that the modulation of certain elements of the PI3K/AKT pathway could help upon the minimization of UPS activation. In fact, it has been shown that AKT can inhibit FOXO activity, potentially acting as an anti-atrophic agent per se [24]. Analysis of the leucine metabolite HMB in the present study also corroborates the concept that minimization of UPS plays a major anti-atrophic role; we have found that although HMB is, similarly to leucine, able to improve PI3K/AKT activity during atrophy, it cannot protect skeletal muscle against loss of mass and function. Likewise, HMB is unable to minimize UPS activation during atrophy.

In summary, we have shown that HMB has no role in protecting skeletal muscle atrophy in the immobilization and dexamethasone models. Leucine however, provides a strong anti-atrophic effect in the immobilization model, and these effects are probably independent of leucine conversion to HMB. It would be of interest in future studies to compare the protective effects of leucine in other models involving skeletal muscle atrophy, such as cancer, AIDS and renal failure.
