# Correction: Leucine and HMB Differentially Modulate Proteasome System in Skeletal Muscle under Different Sarcopenic Conditions

**DOI:** 10.1371/annotation/9060434b-c1df-4d52-8cda-88b9fbfaea51

**Published:** 2013-10-29

**Authors:** Igor L. Baptista, Willian J. Silva, Guilherme G. Artioli, Joao Paulo L. F. Guilherme, Marcelo L. Leal, Marcelo S. Aoki, Elen H. Miyabara, Anselmo S. Moriscot

There was an error in the third affiliation. It should read: School of Arts, Sciences and Humanities, University of Sao Paulo, Sao Paulo, Brazil

